# A New Hyaluronic Emulgel of Hesperetin for Topical Application—An In Vitro Evaluation

**DOI:** 10.3390/jfb15040089

**Published:** 2024-04-01

**Authors:** Raquel Taléns-Visconti, Yousra Belarbi, Octavio Díez-Sales, Jesus Vicente de Julián-Ortiz, Ofelia Vila-Busó, Amparo Nácher

**Affiliations:** 1 Department of Pharmacy and Pharmaceutical Technology and Parasitology, Faculty of Pharmacy and Food Sciences, University of Valencia, Av. Vicent Andrés Estellés s/n, 46100 Valencia, Spain; belarbiyousra31@gmail.com (Y.B.); octavio.diez@uv.es (O.D.-S.); amparo.nacher@uv.es (A.N.); 2 Instituto Interuniversitario de Investigación de Reconocimiento Molecular y Desarrollo Tecnológico (IDM), Universitat Politècnica de València, Av. Vicent Andrés Estellés s/n, 46100 Valencia, Spain; 3 Molecular Topology and Drug Design Research Unit, Department of Physical Chemistry, Faculty of Pharmacy and Food Sciences, University of Valencia, Av. Vicent Andrés Estellés s/n, 46100 Valencia, Spain; jesus.julian@uv.es; 4 Colloids Research Unit, Department of Physical Chemistry, Faculty of Pharmacy and Food Sciences, University of Valencia, Av. Vicent Andrés Estellés s/n, 46100 Valencia, Spain; ofelia.vila@uv.es

**Keywords:** flavonoid, hesperetin, emulgel, hyaluronic acid, stability, topical application, sustained release kinetics

## Abstract

The present study aimed to formulate and characterize a hesperetin formulation to achieve adequate deposition and retention of hesperetin in the epidermis as a target for some cosmetic/dermatological actions. To derive the final emulgel, various formulations incorporating different proportions of Polysorbate 80 and hyaluronic acid underwent testing through a Box–Behnken experimental design. Nine formulations were created until the targeted emulgel properties were achieved. This systematic approach, following the principles of a design of experiment (DoE) methodology, adheres to a quality-by-design (QbD) paradigm, ensuring a robust and purposeful formulation and highlighting the commitment to a quality-driven design approach. The emulsions were developed using the phase inversion method, optimizing the emulgel with the incorporation of hyaluronic acid. Physically stable optimized emulgels were evaluated for their globule size, surface charge, viscosity, pH, electrical conductivity, and hesperetin content. These assays, along with the temperature swing test, were used to select the optimal formulation. It was characterized by a droplet size, d[4,3], of 4.02 μm, a Z-potential of −27.8 mV, an O/W sign, a pH of 5.2, and a creamy texture and proved to be stable for at least 2 months at room temperature. Additionally, in vitro release kinetics from the selected emulgel exhibited a sustained release profile of hesperetin. Skin assays revealed adequate retention of hesperetin in the human epidermis with minimum permeation. Altogether, these results corroborate the promising future of the proposed emulgel in cosmetic or dermatological use on healthy or diseased skin.

## 1. Introduction

The topical route of administration has a long history, but contemporary advancements in methods and technologies have driven its resurgence [1,2]. This mode of administration, whether for cosmetic [3] or dermatologic [4,5] purposes, enjoys widespread utilization across both healthy and diseased skin.

Diverse forms—solid, semi-solid, or liquid—exist for topical applications, spanning powders, creams, ointments, gels, lotions, solutions, and others. In this context, emulsions are one of the most popular physicochemical forms used in cosmetics and pharmaceutical products, e.g., creams, balms, masks, etc. can be presented in the form of an emulsion. Additionally, the emergence of combined systems comprising multiple classical formulations represents a burgeoning field, albeit they are less prevalent in the market despite their high acceptability. Notably, emulgels stand out as captivating and challenging products to explore [6,7,8,9]. These systems arise from the fusion of classic gels with emulsions, resulting in highly functional yet complex dosage forms. Consequently, the gel component extends the formulation’s residence time on the skin by augmenting the viscosity [10]. Simultaneously, the emulsion part shields the active ingredients from degradation and hydrolysis while enhancing skin permeation [11]. Thus, achieving optimal outcomes relies on both retaining higher concentrations of the active ingredient for a sufficient duration and facilitating its effective penetration through the skin [8,12,13,14].

In addition, the exploration of novel formulations, such as emulgels, coincides with the search for effective delivery systems. These intricate systems, which bring together the characteristics of gels and emulsions, underline the persistent drive to optimize topical drug delivery. Moreover, the exploration of natural compounds, particularly polyphenols derived from various food sources, resonates with the ongoing quest for effective dermal preparations. These compounds, renowned for their diverse pharmacological activities, have emerged as promising alternatives to synthetic chemicals in both medical and cosmetic applications. Their intrinsic properties, such as antioxidant, anti-inflammatory, and antibacterial attributes, hold significant potential in mitigating conditions linked to free radical accumulation and skin-related pathologies. However, despite their remarkable bioactivities, the widespread application of polyphenols in medicine and cosmetics faces challenges due to their limited solubility in water. Addressing these formulation limitations becomes pivotal, especially considering the necessity for innovative delivery strategies to harness the full potential of polyphenols in dermal applications [15].

**Figure 1:**
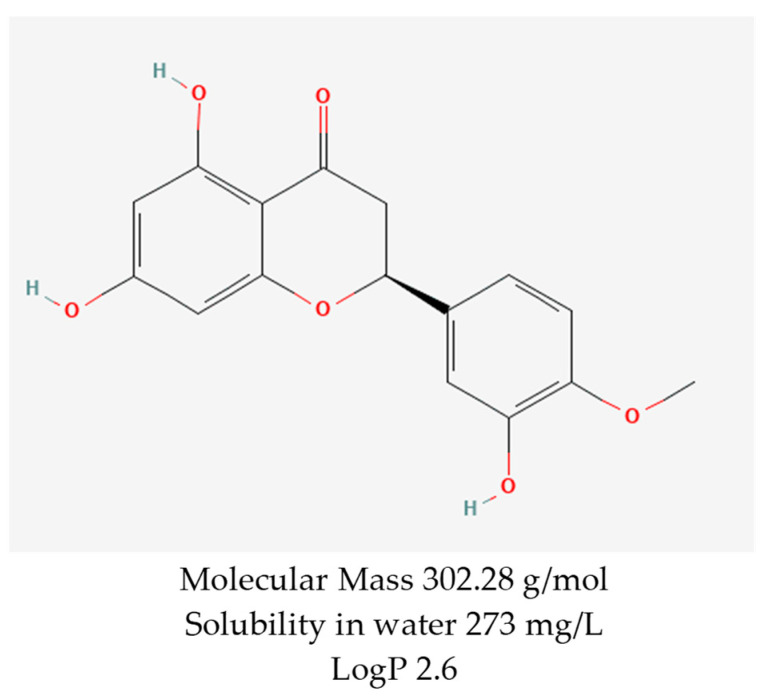
Chemical structure and some properties of hesperetin. Reprinted from Ref. [18].

Notably, hesperetin, a flavanone identified as 3,5,7-trihydroxy-4-methoxyflavanone (C_16_H_14_O_6_) (Figure 1), is the aglycone of hesperidin. It is obtained from the mesocarp (middle layer of the pericarp of the fruit) of the peel of *Citrus sinensis* (sweet orange) and has different types of pharmacological activities, including some that are very favorable for topical administration [16,17].

Hesperetin is primarily acknowledged for its antioxidant, anti-inflammatory, antibacterial, and immunomodulatory properties [19,20], presenting the potential to address conditions associated with free radical accumulation. Furthermore, it demonstrates anticarcinogenic and neuroprotective effects [20,21,22,23]. Additionally, hesperetin has showcased the ability to absorb ultraviolet radiation, offering skin protection and influencing collagen biosynthesis in dermal fibroblasts [24,25]. However, despite these impressive attributes, hesperetin’s utility in medicine and cosmetics has been hindered by its insolubility in water.

Within this context, this study aims to devise an effective topical formulation that ensures adequate deposition and retention of hesperetin within the epidermis.

## 2. Materials and Methods

### 2.1. Materials

Hesperetin (>95% purity) obtained from Sigma-Aldrich (Steinheim production, Steinheim, Germany) was used as the active ingredient. The semi-solid vehicle was prepared using the following compounds: Phospholipid S75 (Lipoid^®^, Abaran Materias Primas S.L., Madrid, Spain), Almond oil (Acofarma^®^, Barcelona, Spain), and Transcutol CG (Gattefossé^®^, Neuilly-Sur-Seine, France). Polysorbate 80, tocopherol, cetostearyl alcohol, propylene glycol, ethanol, methylparaben, propylparaben, and hyaluronic acid of low molecular weight were obtained from Guinama^®^ (Valencia, Spain), and MilliQ water was also used.

### 2.2. Hesperetin Quantification

The hesperetin content was quantified using a reverse phase HPLC method employing a Perkin Elmer^®^ Series 200 HPLC equipped with a UV detector (Perkinelmer España S L, Madrid, Spain), detecting the molecule’s absorbance at 288 nm. The analysis utilized a Teknokroma^®^ Brisa “LC2″ C18 column (150 × 4.6 mm, 5.0 μm) (Teknokroma, Barcelona, Spain) and a mobile phase consisting of a methanol: water MilliQ mixture in a 70:30 proportion, respectively. The flow rate was maintained at 1 mL/min and the sample injection volume was 20 µL. Hesperetin was eluted after 3.5 min and the method was linear in the concentration range of 0.24–62.7 µg/mL with a correlation coefficient of 0.9999. The limits of detection (*LOD*, Equation (1)) and quantification (*LOQ*, Equation (2)) were estimated using the calibration curve procedure, as follows:

(1) $$LOD=\frac{3.3\cdot SD}{S}$$

(2) $$LOQ=\frac{10\cdot SD}{S}$$

where *SD* and *S* are the standard deviation of the y-intercept and the slope of the linear calibration curve, respectively.

Under these conditions, the limit of quantification was 0.918 µg/mL and the limit of detection was 0.303 µg/mL.

### 2.3. Preformulation Studies

#### 2.3.1. Experimental Design

To obtain the final emulgel to be used, several formulations incorporating different proportions of Polysorbate 80 and hyaluronic acid were designed using a Box–Behnken experimental design [26] using the STATISTICA program (v 8.0) [27].

#### 2.3.2. Preparation of Formulations

To obtain the final emulgel to be used during this study, nine formulations with three distinct proportions of Polysorbate 80 (2, 3.5 and 5% *w*/*w*) and hyaluronic acid (0.5, 1, and 1.5% *w*/*w*) were tested (Table 1).

The procedure followed for the preparation of the formulations consisted of phase inversion and subsequent homogenization. In a thermostatic bath, the ingredients of the oil phase and the aqueous phase were heated separately to 75 °C. The aqueous phase was then slowly poured over the oil phase under continuous and vigorous stirring to form emulgels, which were cooled to room temperature. Cetostearyl alcohol added to the oil phase and hyaluronic acid added to the aqueous phase were included to stabilize the formulations. In order to ensure the smallest possible uniform droplet size, a homogenization process using an Ultraturrax^®^ (Janke & Kunkel, IKA-Labortechnik, Staufen, Germany) was completed for 5 min at 24,000 rpm. A hesperetin dispersion of 0.5% *w*/*v* was then added in a sufficient amount of ethanol. The prepared formulations were stored in tightly closed containers and kept at room temperature until testing.

#### 2.3.3. Characterization of Emulgels and Stability Assessment

- Physical Evaluation

The developed formulations were visually and physically inspected for appearance, color, odor, phase separation, pH, electrical conductivity, and viscosity. For pH measurement, a 10% *w*/*v* dispersion of the emulgels was prepared in distilled water and measured using a calibrated Hanna^®^ (Hanna Instruments Ltd., Leighton Buzzard, UK) pH meter. A conductivity meter (Hanna^®^, Hanna Instruments Ltd., Leighton Buzzard, UK) was employed to validate the presence of a continuous aqueous phase, confirming that the emulgels were O/W type. Viscosity was measured using a Brookfield viscometer (ViscoStar plus R.V5002, Fungilab, I.C.T, S.L, Lardero, Spain) at room temperature and with a fixed rotation speed (60 rpm) using an R6 spindle. Centrifugation at 3500 rpm for 30 min determined the emulgels’ stability regarding phase separation, cracking, or creaming (See Table 2). All the measurements were performed in triplicate.

- Optical Microscopy

Microscopic observations of each formulation were carried out using a Carl Zeiss (ScienceServices GmbH company, München, Germany) model GSZ stereoscopic microscope in order to observe the homogeneity of the formulation and to qualitatively verify whether coalescence or an increase in drop size occurred over time.

- Evaluation of Droplet Size and Zeta Potential

The droplet size was measured using a Mastersizer 2000^®^ (Malvern Instruments, Worcestershire, UK). The value used was d[4,3], which corresponds to the weighted average of the volume of the emulgel droplets [28,29]. These data were counterbalanced with size scatter plots. Additionally, the Z-potential was assessed using a Zetasizer nano-S (Malvern Instruments, Worcestershire, UK), with three measurements per sample.

- Temperature Swing Tests

The formulations were subjected to swing tests following two temperature cycles. One of these cycles corresponded to the temperature changes observed in extreme hot–cold conditions such as summer–winter changes, and the other cycle referred to the temperature changes that would occur if the product was frozen and thawed. For this, the samples were stored successively at a temperature of 4 °C and 40 °C for 48 h at each temperature, which was considered one cycle. On the other hand, the respective formulations were stored for 48 h at −20 °C in the deep freezer and at room temperature (25 ± 2 °C), which was considered one freeze–thaw cycle. These procedures were repeated for three cycles each (Table 2). Once the three stages were completed, the samples were subjected to visual, physical, and microscopic evaluation, as described above. In addition, the droplet size, zeta potential, and hesperetin concentration were also measured.

These tests served as selection criteria to identify the stable optimized formulation for subsequent experiments.

- Long-Term Stability Study

The chosen optimized formulation underwent a 2-month long-term stability study at room temperature. After this period, the samples were subjected to physical, visual, and microscopic evaluation, as described above. In addition, the droplet size, zeta potential, and hesperetin concentration were also measured.

### 2.4. In Vitro Hesperetin Release and Skin Retention Studies

The release and skin permeability assessments of hesperetin were conducted on the selected emulgel using a Franz vertical cell (effective diffusion area: 0.785 cm^2^) with a sample size of *n* = 3. The assembled cell was placed in a thermostatic bath at 37 ± 1 °C and maintained under constant agitation. For this purpose, the ‘infinite dose’ condition was used to determine the release and skin permeation coefficients. Briefly, the experiment consisted of adding 500 mg of the test formulation to the donor chamber and a 50% (*v*/*v*) hydroalcohol mixture to the receptor chamber. In the release tests, a cellulose acetate membrane separated the chambers, while the human epidermis was used for the skin permeability tests. The human epidermis was abdominal female skin, which was obtained from patients after they signed informed consent for inclusion before participating in the study. The study was conducted in accordance with the Declaration of Helsinki, and the protocol was approved by the Ethical Committee of the University of Valencia (protocol number: H 1540295606992, approved date: 8 November 2018). The epidermal membranes were obtained via heat separation and immersed in water at 60 °C for 60 s [30] or frozen and stored after fatty tissue removal.

Samples of 200 µL were collected from the receptor chamber, and an equal volume was replenished at different time intervals for up to 24 h. Simultaneously, the accumulated hesperetin in the skin was quantified after methanol extraction. To achieve this, after the 24-h period, the epidermis was cut, placed into a glass vial containing methanol, and sonicated for 2 min to ensure the complete extraction of hesperetin. The resulting dispersion underwent centrifugation at 3000 rpm for 10 min. Subsequently, the supernatant was filtered, and the hesperetin content was measured using HPLC.

The in vitro release kinetics of hesperetin from the emulgel were modeled mathematically. For this purpose, the data were fitted to zero-order (Equation (3)), first-order (Equation (4)) and Higuchi (Equation (5)) equations.

(3) $$Q_{t}=K_{0}t$$

(4) $$Q_{t}=Q_{0}\cdot e^{-k1t}$$

(5) $$\frac{Q_{t}}{Q_{\infty }}=K_{h}\cdot t^{0.5}$$

where t is time, *Q_t_* is the amount of hesperetin released at time *t*, *Q*_0_ is the initial amount of hesperetin, *Q_∞_* is the amount of hesperetin released at time *∞*, and *K*_0_, *K*_1_, and *K_h_* are the hesperetin release rate constants of each of the kinetics.

To determine the mechanism of hesperetin release, Korsmeyer–Peppas (Equation (6)) and Peppas–Sahlin (Equation (7)) mathematical models were employed and fitted to the experimental data using Sigmaplot 10.0^®^ (Systat Software, Inc., San Jose, CA, USA).

(6) $$\mathrm{Korsmeyer}-\mathrm{Peppas}:\;\frac{Q_{t}}{Q_{\infty }}=K\cdot t^{n}$$

(7) $$\mathrm{Peppas}-\mathrm{Sahlin}:\;\frac{Q_{t}}{Q_{\infty }}=K_{1}t^{n}{+K}_{2}t^{2n}$$

where *Q_t_/Q_∞_* represents the fraction released of the active ingredient, *K*, *K*_1_, and *K*_2_ are the diffusion constants, *t* is the time in which the active substance is released, and *n* is the exponent that characterizes the diffusion process. So for *n* < 0.5, the release is Fickian, between 0.5 < *n* < 1.0, it indicates that an anomalous process has occurred, while for *n* = 1, the release obeys zero-order kinetics [31].

Additionally, for the skin permeation studies, as a representative of the diffusion process in the experimental conditions, the Scheuplein equation was used, which relates the quantities (*Q*, mg/cm^2^) permeated with time (*t*, hours) (Equation (8)):

(8) $$Q_{\left(t\right)}=A\cdot P\cdot L\cdot C\cdot \left\lfloor D\cdot \frac{t}{L^{2}}-\frac{1}{6}-\frac{2}{\pi^{2}}\cdot \sum_{n=1}^{\infty }\frac{{\left(-1\right)}^{n}}{n^{2}}\cdot Exp\left(\frac{-D\cdot n^{2}\cdot \pi^{2}\cdot t}{L^{2}}\right)\right\rfloor$$

where *A* is the useful diffusion area (cm^2^), *P* is the distribution coefficient of the drug between the skin and the donor vehicle, *L* is the thickness of the membrane (cm), *C* is the drug concentration in the donor solution (mg/mL), *D* is the diffusion coefficient in the membrane (cm^2^/h), and *n* is a value integer between one and infinity.

Equation (8) has been simplified to Equation (9) and represents the linear section of the percutaneous absorption process:

(9) $$Q_{\left(t\right)}=A\cdot P\cdot L\cdot C\cdot \left\lfloor D\cdot \frac{t}{L^{2}}-\frac{1}{6}\right\rfloor$$

The accumulated hesperetin quantities against time were fitted to Equation (9). The values of *P* and *D* were obtained as the primary parameters of the fit. From these values, the latency time (*t_L_*, h), the permeability coefficient (*K_p_*, cm/h), and the flow (*J*, µg/cm^2^·h) were calculated:

(10) $$t_{L}=\frac{1}{6\cdot D}$$

(11) $$K_{p}=\frac{P\cdot D}{L}$$

(12) $$J=K_{p}\cdot C$$

### 2.5. Statistical Analysis

To observe whether there were significant differences between the formulas tested, statistical tests were performed using the IBM SPSS 22.2 Statistics program (Systat Software, Inc., San Jose, CA, USA) with a fixed level of significance of *p* = 0.05. Initially, the Levene statistic test was employed to ascertain the homogeneity of variances in the results. Subsequently, for the cases meeting this condition, the parametric Scheffe’s multiple comparison test was applied, while the non-parametric Dunnett T3 test was utilized for the cases not meeting the homogeneity condition. The results are presented as mean values alongside their standard deviations.

## 3. Results and Discussion

The aim of this work was to provide an effective formulation strategy for enhanced skin retention of a highly lipophilic molecule, such as hesperetin, to have the potential to provide topical formulations for the skin. In that context, a hyaluronic emulgel could be the ideal vehicle, as an emulsion allows for the incorporation of hydrophilic/lipophilic substances due to its biphasic nature, and hyaluronic acid permits the retention of active substances longer on the skin, prolonging their action [32]. Additionally, semi-solid formulations prepared using this polymer are characterized by excellent sensory and rheological properties and have shown excellent qualities as wound-healing formulations [33,34].

### 3.1. Experimental Design

The Box–Behnken experimental design encompassed three distinct proportions of Polysorbate 80 and hyaluronic acid. The design, as detailed in Table 3, comprised nine trials (three different variable levels applied to each component).

Wherein:

V1 levels of Polysorbate 80 concentrations are represented as follows:

- (−1) = 2.0% *w*/*w*
- (0) = 3.5% *w*/*w*
- (+1) = 5.0% *w*/*w*

V2 levels of hyaluronic acid concentrations are depicted as:

- (−1) = 0.5% *w*/*w*
- (0) = 1.0% *w*/*w*
- (+1) = 1.5% *w*/*w*

The specific values for each level were determined based on prior formulation experiences, contributing to the assignment of these variable levels.

### 3.2. Physical Evaluation

**Figure 2:**
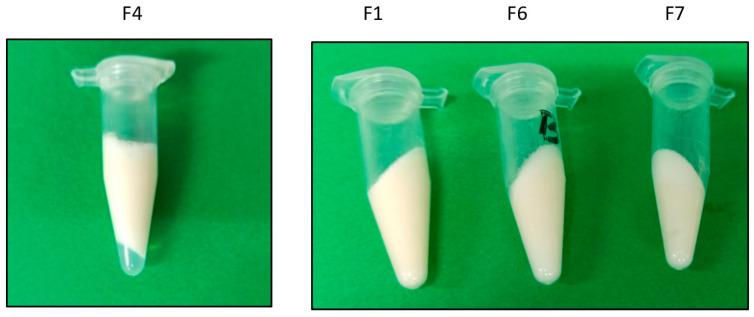
Representative images of results of centrifugation assays (3500 rpm/30 min) in different formulations (F4, showing phase separation; F1, F6, and F7, showing stability).

All the developed emulgels (F1–9) were smooth, and most of them were creamy in texture without clumps, with a white color in appearance and a pleasant odor. The pH values were observed to be 5.2–6.3. Moreover, all pH values were around that of the skin pH (4.5–6), suggesting an acceptable and non-irritating value [34]. Moreover, the creamy formulations that did not show phase separation were those with a higher hyaluronic acid concentration (1.5% *w*/*v*) with a viscosity of around 3020 ± 7.4 mPa·s (Figure 2). Finally, the electrical conductivity showed values of 430–585 µS thus confirming that all the formulations were of an external aqueous phase.

### 3.3. Optical Microscopy

**Figure 3:**
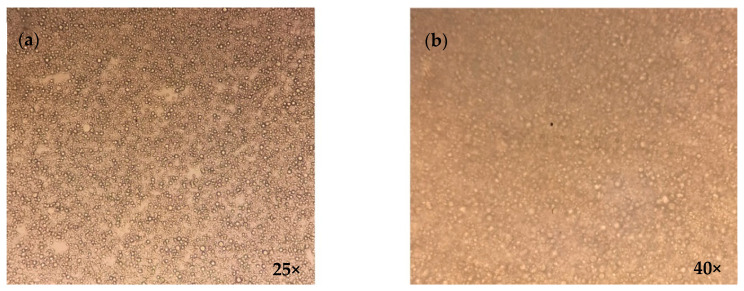
Representative image of emulgel formula F7 obtained using Carl Zeiss stereo microscope model GSZ at 25× (**a**) and 40× (**b**).

The different formulations were observed under the visual microscope at zero time as well as after the cycle tests. In addition, the formulation that showed the best results (F7) was also observed at one and two months (long term). Homogeneity and the absence of aggregates were verified. A representative image is shown in Figure 3.

### 3.4. Temperature Swing Test

The formulations that did not show flocculation, creaming, or cracking during physical evaluation were subsequently subjected to the temperature swing test. After the three cycles of temperature changes, the formulations were characterized following the analyses mentioned in the methodology section, thus allowing for observation of the changes produced in the emulgels. The results before and after the temperature tests showed that the values of the surface charge, droplet size, pH, conductivity, and hesperetin content (%) were more stable and consistent for formulation 7. A representative image of emulgel formula F7 obtained using a Carl Zeiss stereo microscope model GSZ is shown in Figure 3. Additionally, a control emulgel was used with the same composition but without hesperetin to verify that the introduction of this molecule did not modify any characteristics (Table 4).

**Figure 4:**
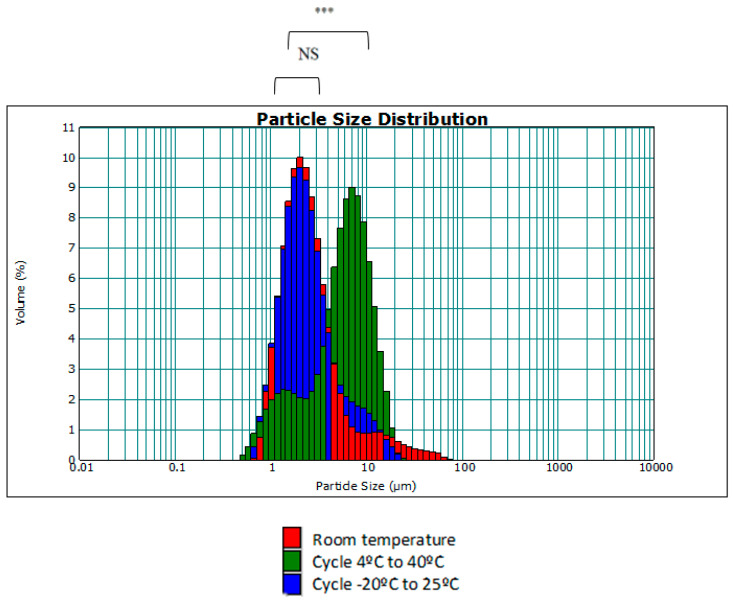
Drop size distribution (d[4,3]) in accelerated temperature tests (NS: no significant difference; ***: *p* < 0.001).

Moreover, the size distribution of the droplets was evaluated under these temperature changes with respect to the same emulgel at room temperature (initial state). The aim was to investigate whether the droplets underwent a change in size with respect to temperature. The plots obtained using the MasterSizer showed the particle size distribution. Figure 4 shows the particle size distribution of the F7 emulgel and the distribution after temperature cycling.

As seen in Figure 4, with increasing temperature, the droplets undergo a slight increase in size as the size dispersion curve of the 4 °C to 40 °C cycle has shifted slightly to the right. This may be due to the fact that the affinity of surfactants varies with temperature, resulting in some degree of aggregation of smaller droplets. Although there is statistical significance between the 4 °C to 40 °C cycle curve and the rest of the results, these differences have no practical repercussion and will not influence the quality of the cosmetic cream to be developed, since the range of measurements is still between 0.2–12 μm, as in the case of room temperature and the −20–25 °C cycle.

**Figure 5:**
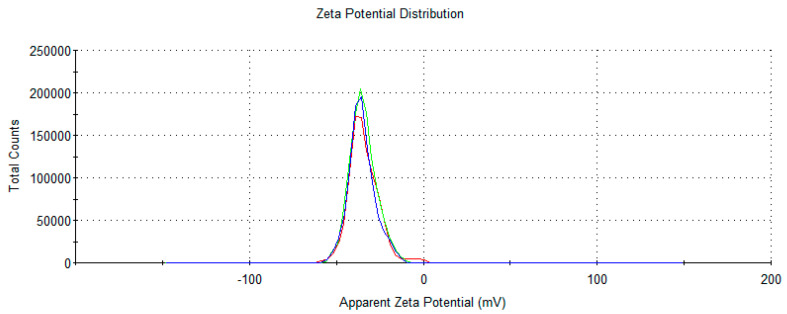
Representative image of Z-potential determination Zetasizer nano-S (Malvern Instruments, Worcestershire, UK).

On the other hand, the formulations are shown to be stable after temperature cycling, as the Z-potential (Figure 5), sign, and pH are not affected, with no statistical significance. Furthermore, preliminary chemical stability tests with the formulations incorporating hesperetin have shown that temperature cycling does not affect their initial concentration. Altogether, this affirms that the emulgel is stable regarding temperature changes; therefore, it may be possible to store it without refrigeration at room temperature without seasonal changes altering the amount of the active ingredient.

### 3.5. Long-Term Stability Study

Due to the satisfactory results of the preliminary studies described in the previous section, the F7 emulgel was subjected to long-term studies. These consisted of measuring the Z-potential, conductivity, pH, droplet size, and quantification of hesperetin for two months, once per month, at room temperature.

**Figure 6:**
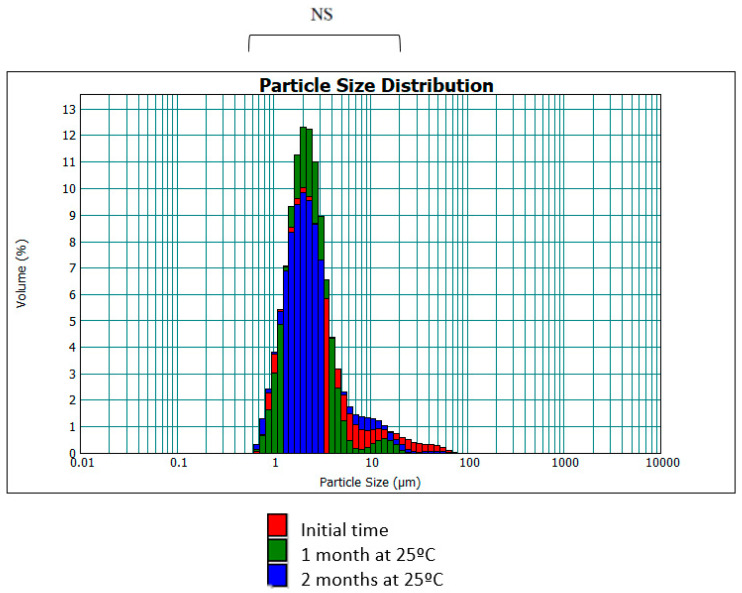
Distribution of droplet size (d[4,3]) as a function of time (NS: no significant differences).

Figure 6 shows the droplet size distribution as a function of time. It can be seen that the majority of droplets are between 0.6–6 μm, which is similar to the size distribution of the formula without hesperetin.

**Figure 7:**
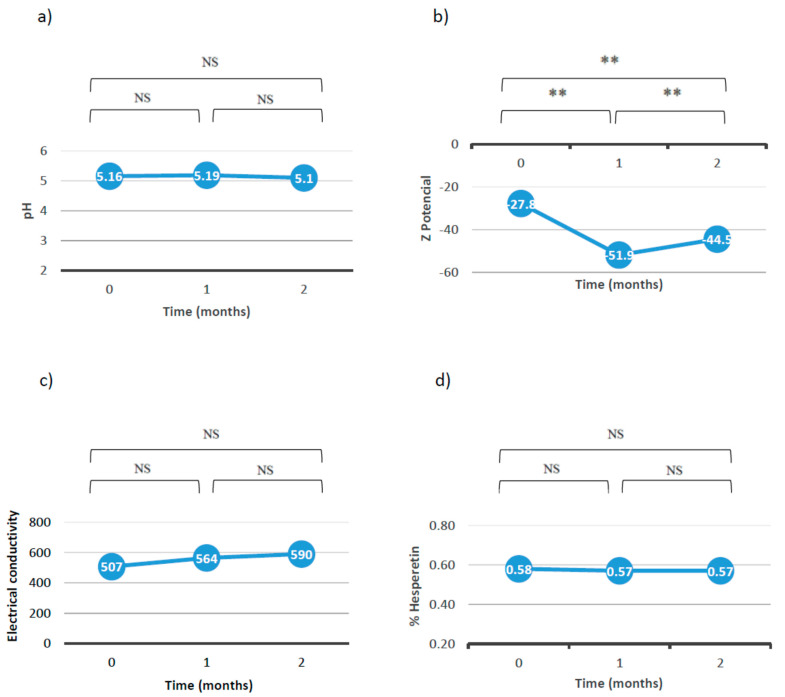
Results of (**a**) pH, (**b**) Z—potential, (**c**) conductivity, and (**d**) percent of hesperetin with respect to the time (NS: no significant differences; **: *p* < 0.01).

Figure 7 shows the values of pH, Z-potential, and conductivity and the percentage of hesperetin with respect to time.

Overall, these results allow us to conclude that the emulgel is stable after 2 months at room temperature since it does not undergo significant variations in terms of pH, conductivity, droplet size, or percentage of the active ingredient. In fact, although significant differences in the Z-potential data were observed, in practice, the emulgel was considered to be stable since it favored the repulsion between the droplets and reduced the tendency to aggregate. On the other hand, the pH remained around 5, which is within the favorable range for topical use. The conductivity remained stable, indicating that the emulgel did not undergo any change in its sign and continued to be O/W. Finally, the percent of hesperetin did not decrease after two months.

### 3.6. Release Studies

The release of a drug is a determining parameter in any pharmaceutical development as the therapeutic efficacy of any drug is dependent upon the release of the drug from the pharmaceutical preparation [35]. To establish the release kinetics of the active ingredient from the emulgel with hesperetin (F7), the diffusion technique in “Franz” type cells was used. In this technique, the vehicle located in the donor compartment is kept in contact with a porous support membrane. This contact allows the free diffusion of drug molecules into the receiving compartment.

**Figure 8:**
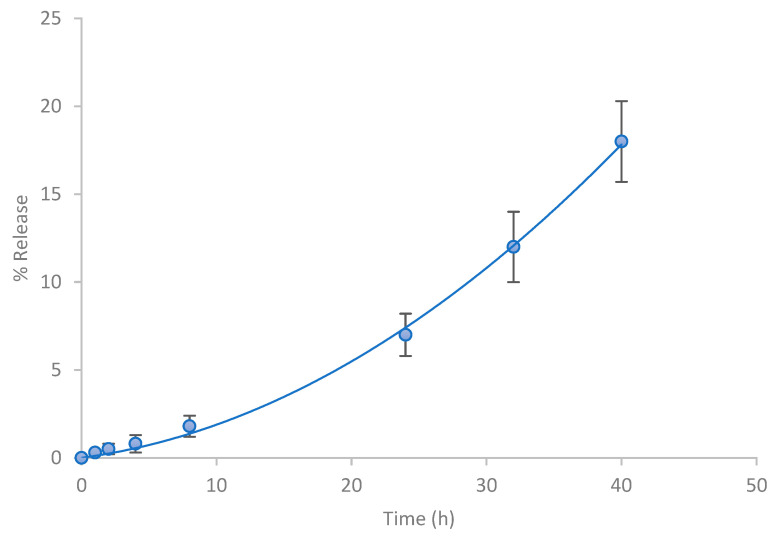
Percentage of hesperetin released as a function of time (h) for the emulgel (*n* = 4).

The percentages of hesperetin released as a function of time from the formulation assayed are expressed in Figure 8. In total, 10% of the active ingredient was released after 24 h for a semi-solid vehicle and approximately 20% was released after 48 h.

To describe the release kinetics of hesperetin, first-order, zero-order, and Higuchi models were tested and compared (Table 5).

According to the results of the release kinetics, the data were best fitted to the zero-order model with the lower sum of squares and Akaike’s criterion values (*K*_0_ = 0.43%/h, r = 0.994). Moreover, the Kosmeyer–Peppas and Peppas–Shalin models were used to characterize and predict the mechanism of hesperetin release from the emulgel. The values of the parameters obtained (Table 6) indicate that both the Peppas–Sahlin and Korsmeyer–Peppas kinetic models can appropriately describe the release profile data. Considering that *K*_2_ is practically equal to zero, it can be concluded that the release process is of zero order for the emulgel (*n* = 1.05). These results agree with those previously shown in Table 5, where the lowest sum of squares and AIC among the tested formulations was a zero-order model and were in accordance with the study of Zillich et al. [36] in which zero-order kinetic models were obtained for hydrophobic polyphenol release with a similar molecular weight to hesperetin from various cosmetic O/W emulgels. The release of actives from topical formulations depends upon several factors including gelling agents, emulsifying agents, and viscosity [37]. It is likely that the hydrophobic molecules, located apparently inside the dispersed lipid phase, had to overcome the interface O/W prior to the diffusion to the release media. Moreover, the controlled release rate in the emulgel could be explained by the fact that the hesperetin-loaded oil droplets were additionally coated by the polymeric surface. These results were in agreement with information found in the literature, which describes the release profile of combined systems [7,38].

### 3.7. Skin Permeation Studies

In order to understand the action of this new vehicle, a permeation study using the human epidermis was carried out by applying an emulgel of hesperetin or a hydroalcohol solution of hesperetin (0.5 mg/mL in both cases) on the skin.

**Figure 9:**
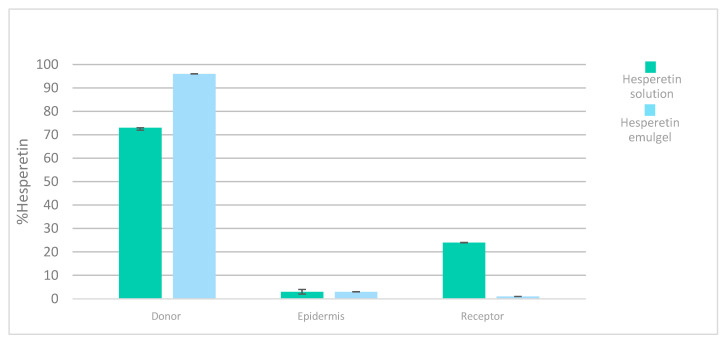
Distribution of hesperetin (%) after 24 h human epidermis permeation experiments after application of a solution and an emulgel, respectively.

The accumulative hesperetin amounts (%) obtained from the different formulations in the donor chamber, epidermis skin, and receptor chamber are shown in Figure 9. As can be seen, the solution provided the greatest penetration rate of hesperetin in the receptor compartment at 24 h compared to that of the hesperetin emulgel. This fact could be attributed to the formulation characteristics, like the higher oil content and viscosity [37], which are in accordance with the zero-order release kinetics previously observed with the emulgel (see Figure 8).

Nevertheless, the accumulation of hesperetin in the epidermis from the solution or from the emulgel occurred in equal amounts, which would mean that the skin was saturated in hesperetin with both formulations (Figure 9).

**Figure 10:**
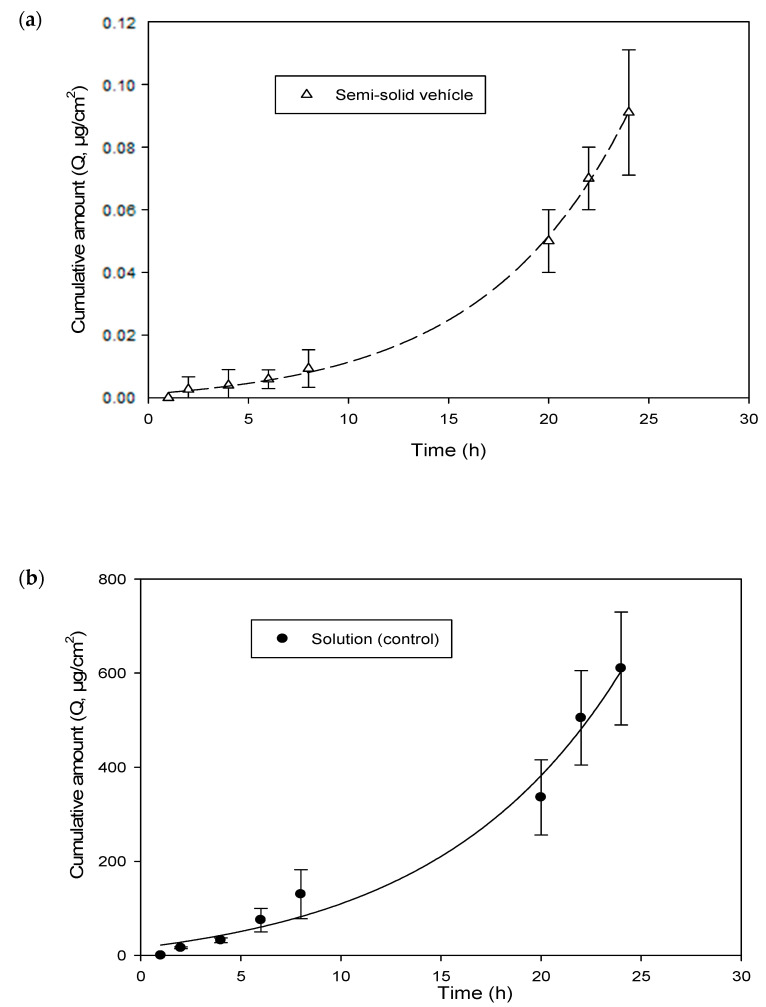
Cumulative amount of hesperetin across human skin. (**a**) Semi-Solid Vehicle. (**b**) Solution (Control).

Figure 10 shows the permeation kinetics of hesperetin (cumulative quantities as a function of epidermis surface (*Q*, μg/cm^2^) versus time) through the human epidermis from the solution and the emulgel, respectively. In addition, the skin-vehicle partition coefficients, P, (Equation (9)), the diffusion coefficients within the epidermis, D (cm^2^/h) (Equation (9)), the latency time, *t_L_* (h) (Equation (10)), the permeability coefficient, *K_p_* (cm/h) (Equation (11)), and the flow, *J* (µg/cm^2^·h) (Equation (12)), were calculated for the solution and the emulgel. These values are presented in Table 7.

In accordance with Table 7, the lag time (*t_L_*) was 2.2 h for the solution and 6.7 h for the emulgel. This indicates that the steady state (constant flow) was reached three times later for the emulgel, as expected.

Significant differences between the flux values and the amount of permeated hesperetin provided by the emulgel preparation and hesperetin solution were detected (*p* < 0.01).

The flux (=24 µg/cm^2^·h) was much higher in the case of the hesperetin solution, which indicates greater penetration of the tested molecule through the epidermis and, therefore, a greater amount in the receptor compartment (Figure 9). A high flow rate is not necessarily better when the objective is for the active ingredient to reach its target layer (in our case, the epidermis). In this case, a slower flow is preferred, and the active ingredient should not reach the systemic circulation since a local action is of interest for the cosmetic effect. In this sense, the emulgel is a preparation that improves the topical application of hesperetin as a dermatological product and exerts greater control over the release and penetration of the active ingredient. (flux = 0.004 µg/cm^2^·h). Moreover, the skin permeation coefficients, *K_p_*, were approximately ten times lower than the respective release coefficients, *K* (Table 6). These observations indicate that the epidermis is a significant barrier against the permeation of hesperetin when the vehicle is the emulgel.

The diffusion coefficients (D, cm^2^/h) did not differ between the control and the emulgel. Nevertheless, the human epidermis-vehicle (P) partition coefficient was much lower for the emulgel. This implies that the skin is saturated sooner (shorter lag time) with the solution. Therefore, the permeability coefficient of the hesperetin showed faster access to the receptor compartment, as would be expected under in vivo conditions.

## 4. Conclusions

Preformulation trials allowed the design and optimization of a formulation in the form of an aqueous external phase emulgel that was shown to have the appropriate characteristics to administer hesperetin topically. The presence of 3.5% *w*/*w* Polysorbate 80 and 1.5% *w*/*w* hyaluronic acid exhibits a stable formulation and a zero-order release of hesperetin, which contributes to achieving appropriate epidermal retention, which is desirable to develop a local action both for dermatological or cosmetical use, for example, in possible vitiligo therapy or solar photoprotection. Overall, the proposed formulation could be an alternative with great potential for the development of topical formulations based on hesperetin.

## Figures and Tables

**Table 1:** Varying composition of emulgel formulations (% *w*/*w*).

Ingredient (% *w*/*w*)	F1	F2	F3	F4	F5	F6	F7	F8	F9
Hesperetin	0.5	0.5	0.5	0.5	0.5	0.5	0.5	0.5	0.5
Phospholipid	5	5	5	5	5	5	5	5	5
Polysorbate 80	2	3.5	2	2	3.5	5	3.5	5	5
Cetostearyl alcohol	3	3	3	3	3	3	3	3	3
Almond oil	10	10	10	10	10	10	10	10	10
Transcutol GC	4	4	4	4	4	4	4	4	4
Tocopherol	0.1	0.1	0.1	0.1	0.1	0.1	0.1	0.1	0.1
Propylene glycol	5	5	5	5	5	5	5	5	5
Methylparaben	0.1	0.1	0.1	0.1	0.1	0.1	0.1	0.1	0.1
Propylparaben	0.05	0.05	0.05	0.05	0.05	0.05	0.05	0.05	0.05
Hyaluronic acid	1.5	0.5	0.5	1	1	1.5	1.5	1	0.5
Ethanol	q.s	q.s	q.s	q.s	q.s	q.s	q.s	q.s	q.s
Water	68.75	68.25	69.75	69.25	67.75	65.75	67.25	66.25	66.75

**Table 2:** Conditions for forced stability studies.

Forced sedimentation test	30 min at 3500 rpm
Temperature swing test	Cold–heat cycle 4 °C to 40 °C
	Freeze–thaw cycle −20 °C to 25 °C

**Table 3:** Coded representations of the actual values for the variables.

Independent Variable	F1	F2	F3	F4	F5	F6	F7	F8	F9
V1: Concentration of Polysorbate 80	−1	0	−1	−1	0	+1	0	+1	+1
V2: Concentration of hyaluronic acid	+1	−1	−1	0	0	+1	+1	0	−1

**Table 4:** Results of characterization and stability of the emulgel.

Emulgel	State	Z-Potential	Droplet Size d[4,3] µm	pH	Conductivity (µS)	% Hesperetin
Control		−28.3 ± 1.39	2.07	5.89	487	-
F7 (selected formula with 0.5% hesperetin)	Initial	−27.8 ± 0.68	4.02	5.16	507	0.58
	Cycle 4/40 °C	−25.6 ± 2.89	6.52	5.13	564	0.56
	Cycle −20/25 °C	−25.8 ± 0.70	3.28	5.36	587	0.56

**Table 5:** Release kinetics from emulgel, statistical summary of the fitted models (SS: sum of squares, AIC: Akaike Information Criterion).

	Zero-Order Kinetics	First-Order Kinetics	Square-Root Kinetics
SS	1.87	64.58	86.77
AIC	5.7S4	27.01	28.78

**Table 6:** Parameters obtained from those adjusted using the equations indicated in the characterization of solid—vehicle formulation from release kinetics for the models used.

Diffusion Model	Parameters	Semi-Solid Vehicle
Korsmeyer–Peppas	*K*	0.10 ± 0.02
	*n*	1.06 ± 0.06
	r	0.9990
Peppas–Shalin	*K*_1_	0.13 ± 0.08
	*K*_2_	0.002 ± 0.004
	*n*	1.05 ± 0.33
	*r*	0.9993

**Table 7:** Parameters obtained for the percutaneous absorption for the solution and semi-solid vehicle (Equation (9)).

Parameter	Solution (Control)	Semi-Solid Vehicle
*P*	648.35 ± 367.67	0.374 ± 0.077
*D* (cm^2^/h)	0.075 ± 0.038	0.025 ± 0.004
*t_L_* (h)	2.2 ± 0.4	6.7 ± 0.7
*K_p_·*10^−3^ (cm/h)	48.7 ± 5.9	0.010 ± 0.002
*J* (μg/cm^2^·h)	24.2 ± 2.8	0.004 ± 0.001
*r*>	0.98	0.96

## Data Availability

The data presented in this study are available on request from the corresponding author.
